# GLUTATHIONE DEFICIENCY AND OXIDATIVE STRESS IN AGING: METABOLIC MECHANISM AND TARGETED INTERVENTION

**DOI:** 10.1093/geroni/igz038.1551

**Published:** 2019-11-08

**Authors:** Farook Jahoor, George E Taffet, Rajagopal V Sekhar

**Affiliations:** Baylor College of Medicine, Houston, Texas, United States

## Abstract

The free-radical theory of aging suggests that age-related functional decline is mediated by increases in free-radical induced oxidative-stress. Cells normally depend on antioxidants for protection against oxidative-stress. Glutathione is the most abundant endogenous intracellular antioxidant protein composed of 3 amino-acids, cysteine, glycine and glutamic-acid, and is known to be deficient in older-humans. We investigated Glutathione kinetics in older humans using a stable-isotope tracer-based approach, and found that compared to younger humans, older-humans had severe Glutathione deficiency as a result of decreased synthesis caused by limited availability of glycine and cysteine, and associated with elevated oxidative-stress. Orally supplementing glycine and cysteine (provided as N-acetylcysteine) at doses of 1.33mmol/kg/d and 0.81mmol/kg/d respectively for 2-weeks corrected their intracellular deficiency, normalized Glutathione synthesis rates and lowered oxidative-stress to levels in younger controls. These results suggest that short-term supplementation of GlyNAC at these doses can successfully correct intracellular Glutathione deficiency in older-humans.

